# Gepotidacin Pharmacokinetics-Pharmacodynamics against Escherichia coli in the One-Compartment and Hollow-Fiber *In Vitro* Infection Model Systems

**DOI:** 10.1128/AAC.00122-21

**Published:** 2021-11-17

**Authors:** Brian D. VanScoy, Elizabeth A. Lakota, Haley Conde, Steven Fikes, Sujata M. Bhavnani, Philippa B. Elefante, Nicole E. Scangarella-Oman, Paul G. Ambrose

**Affiliations:** a Institute for Clinical Pharmacodynamics, Schenectady, New York, USA; b GlaxoSmithKline, Collegeville, Pennsylvania, USA

**Keywords:** *Escherichia coli*, gepotidacin, pharmacokinetics-pharmacodynamics

## Abstract

Gepotidacin is a novel, first-in-class triazaacenaphthylene antibiotic that inhibits bacterial DNA replication by a distinct mechanism of action with an *in vitro* spectrum of activity that includes Escherichia coli. Our objectives herein were the following: (i) to identify the pharmacokinetic-pharmacodynamic (PK-PD) index associated with the efficacy of gepotidacin against E. coli; (ii) to determine the magnitude of the above-described PK-PD index associated with various bacterial reduction endpoints for E. coli; and (iii) to characterize the relationship between gepotidacin exposure and on-therapy E. coli resistance amplification. A 24-h one-compartment *in vitro* infection model was used to investigate the first two study objectives, and a 10-day hollow-fiber *in vitro* infection model was used to evaluate the third objective. For the dose-fractionation studies (objective i) in which E. coli NCTC 13441 (gepotidacin MIC, 2 mg/liter) was evaluated, gepotidacin free-drug area under the concentration-time curve (AUC) from 0 to 24 h to the MIC (AUC/MIC ratio) was identified as the PK-PD index most closely associated with change in bacterial burden (*r^2^* = 0.925). For the dose-ranging studies (objective ii), in which four E. coli isolates (gepotidacin MIC range, 1 to 4 mg/liter) were studied, the magnitude of the median gepotidacin free-drug AUC/MIC ratio associated with net bacterial stasis and 1- and 2-log_10_ CFU reductions for the pooled data set was 33.9, 43.7, and 60.7, respectively. For the hollow-fiber *in vitro* infection model studies (objective iii), in which one isolate (E. coli NCTC 13441; gepotidacin MIC, 2 mg/liter) was evaluated, gepotidacin free-drug AUC/MIC ratios of 275 and greater were sufficient to suppress on-therapy resistance amplification. Together, the data generated from these studies will be useful to support discrimination among candidate dosing regimens for future clinical study.

## INTRODUCTION

Gepotidacin is a novel, first-in-class triazaacenaphthylene antibiotic that inhibits bacterial DNA replication by a distinct mechanism of action (1, 2), which confers activity against most strains of Escherichia coli and Staphylococcus saprophyticus, including those resistant to current antibiotics (3–5). Gepotidacin binds to the same bacterial target enzymes (e.g., DNA gyrase and topoisomerase IV) as fluoroquinolone antimicrobial agents. However, gepotidacin does so in a manner that is different than other agents targeting these enzymes (5), and it retains its *in vitro* activity against most fluoroquinolone-resistant isolates (6).

We have previously explored gepotidacin’s pharmacokinetic-pharmacodynamic (PK-PD) profile against Staphylococcus aureus, Streptococcus pneumoniae, and Neisseria gonorrhoeae (7, 8). Most recently, we explored the relationship between gepotidacin exposure and the time course of N. gonorrhoeae drug resistance amplification in a hollow-fiber *in vitro* infection model (8). We successfully identified magnitudes of gepotidacin exposure and dosing schedules sufficient to suppress on-therapy resistance amplification (e.g., single 4.5- to 12-g doses or 6 g divided into two equal quantities administered 8 to 12 h apart). One such gepotidacin dosing regimen (6 g administered in two equally divided quantities 6 to 12 h apart) has been carried forward for an uncomplicated urogenital gonorrhea phase 3, randomized, multicenter, comparative clinical trial that is currently ongoing (9).

Herein, we described *in vitro* studies undertaken to explore the PK-PD of gepotidacin against E. coli. The studies undertaken had the following three main objectives: (i) to identify the PK-PD index associated with the efficacy of gepotidacin against E. coli; (ii) to determine the magnitude of the PK-PD index associated with various bacterial reduction endpoints for E. coli; and (iii) to characterize the relationship between gepotidacin exposure and on-therapy resistance amplification.

## RESULTS

### *In vitro* susceptibility testing.

Gepotidacin and meropenem MIC values for each challenge isolate are shown in Table 1. Gepotidacin MIC values in Muller-Hinton broth ranged from 1 to 4 mg/liter, while that for meropenem varied from 0.03 to >8 mg/liter. Gepotidacin and meropenem MIC values determined using agar dilution methodologies were similar to those identified using broth dilution methodologies.

**TABLE 1:** Gepotidacin and meropenem broth microdilution and agar dilution MIC values for each of the E. coli challenge isolates

Isolate	Resistance mechanism	MIC (mg/liter)					
		Broth microdilution			Agar dilution		
		Gepotidacin	Meropenem	Levofloxacin	Gepotidacin	Meropenem	Levofloxacin
EC-NCTC-13441	CTX-M-15 (ST-131)	2	0.03	16	1	0.06	16
EC-ALL	NDM-1, CTX-M-15, OXA-1, OXA-2	4	0.06	32	2	0.06	32
EC-25922	Wild type	1	0.03	0.015	1	0.03	0.015
EC-IR5-3257	NDM-1, CTX-M-1/15	4	>8	32	2	>8	32

### Frequency of resistance studies.

The frequency of resistance for each challenge E. coli isolate to gepotidacin is presented in Table 2. The frequency of resistance was low across all four challenge isolates. The highest frequency of resistance (4.5 × 10^−9^) was observed with isolate EC-IR5-3257 at 2.5 times the gepotidacin baseline MIC. When a gepotidacin mutant was identified (isolates EC-IR5-3257 and 13441), its gepotidacin MIC value was 2- to 4-fold higher than its corresponding baseline MIC. Each mutant gepotidacin MIC value was restored to its matching baseline value by a broad-spectrum efflux pump inhibitor.

**TABLE 2:** Frequency of resistance and mutant susceptibility test results from plates containing 2.5 or 4 times the gepotidacin baseline MIC for each of the E. coli challenge isolates

Isolate	Inoculum (CFU/ml)	Frequency of resistance at 48 h		Gepotidacin MIC of resistant isolate (mg/liter)
		2.5× MIC	4× MIC	
EC-13441	6.4 × 10^8^	1.1 × 10^−9^	<1.6 × 10^−9^	4 to 8
EC-ALL	8.3 × 10^8^	<1.2 × 10^−9^	<1.2 × 10^−9^	NA^*a*^
EC-25922	8.9 × 10^8^	<1.1 × 10^−9^	<1.1 × 10^−9^	NA
EC-IR5-3257	6.4 × 10^8^	4.5 × 10^−9^	<1.6 × 10^−9^	8 to 16

a NA, not applicable.

### Pharmacokinetic studies.

**FIG 1:**
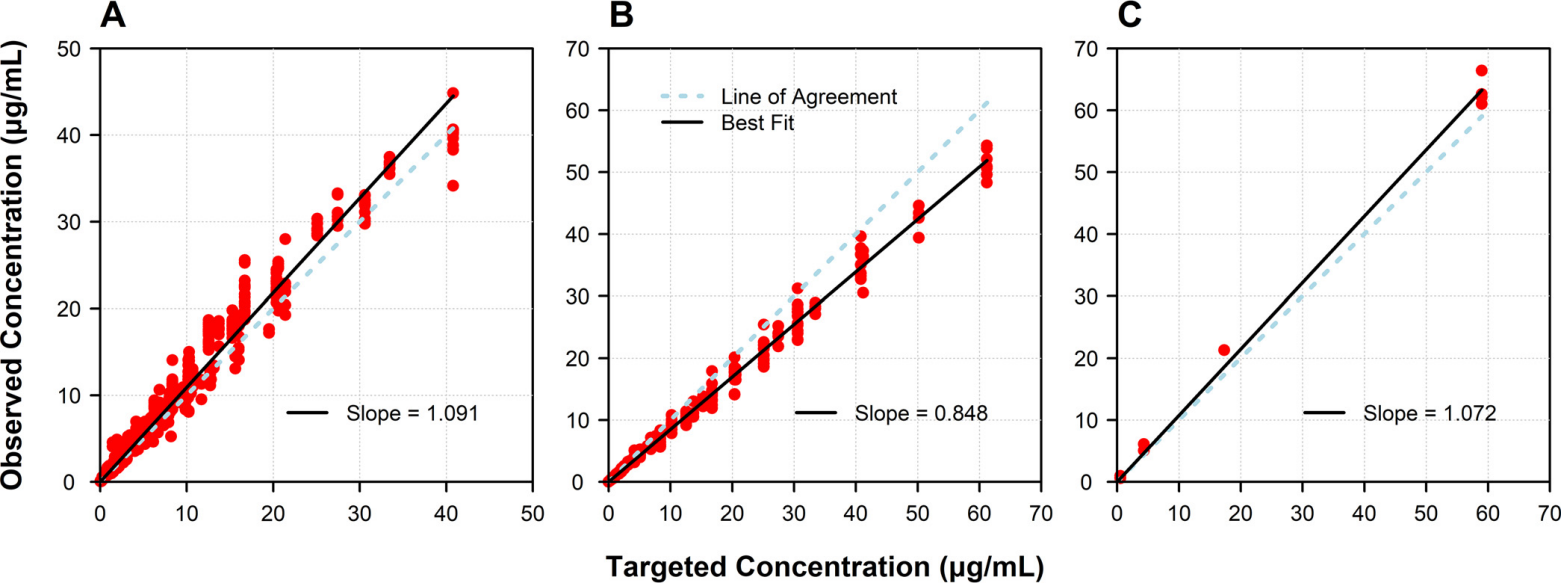
Relationships between observed and targeted gepotidacin concentrations evaluated in a one-compartment *in vitro* infection model (A) and gepotidacin and meropenem concentrations evaluated in a hollow-fiber *in vitro* infection model (B and C, respectively).

The targeted gepotidacin and meropenem concentration-time profiles were simulated well within the one-compartment and hollow-fiber *in vitro* infection models. In each instance, the coefficient of determination (*r*^2^) and slope approached 1 with intercepts approaching 0 (Fig. 1).

### Dose-fractionation studies.

**FIG 2:**
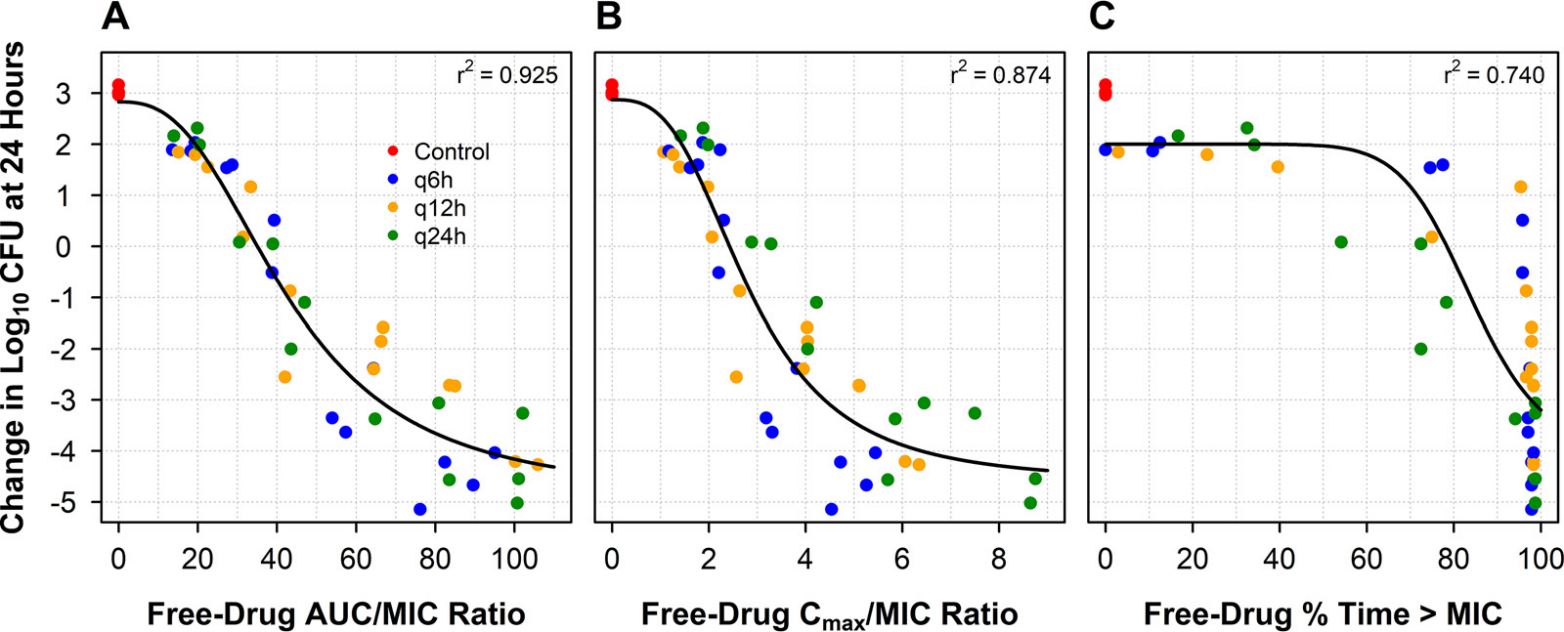
Relationships between change in log_10_ CFU/ml from baseline at 24 h and gepotidacin free-drug AUC/MIC ratio (A), *C*_max_/MIC ratio (B), and %*T*>MIC (C) based on data from the dose-fractionation studies for E. coli NCTC 13441 carried out using a one-compartment *in vitro* infection model.

The results of the dose-fractionation studies carried out in the one-compartment *in vitro* infection model are shown in Fig. 2. As evidenced by high *r*^2^ values (0.925) and the data dispersion around the fitted function, gepotidacin free-drug AUC/MIC ratio best described the relationship between change in log_10_ CFU/ml from baseline at 24 h and gepotidacin exposure indexed to MIC. The Hill-type model parameter estimates (standard error) describing the change in log_10_ CFU/ml from baseline at 24 h and free-drug AUC/MIC ratio were the following: change in log_10_ CFU/ml from baseline without drug (*E*_0_), 2.83 (0.34); maximal change in log_10_ CFU/ml from baseline (*E*_max_), 7.74 (0.88); Hill coefficient, 2.66 (0.57); and free-drug AUC/MIC ratio associated with half-maximal effect (EC_50_), 43.1 (3.92).

### Multiple-isolate dose-ranging studies.

**FIG 3:**
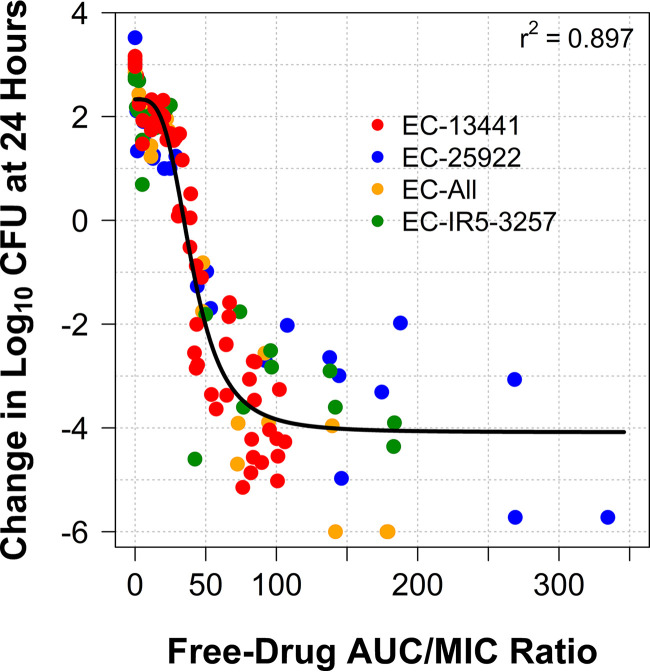
Relationship between change in log_10_ CFU/ml from baseline at 24 h and gepotidacin free-drug AUC/MIC ratio based on data from the dosing-ranging studies for four E. coli challenge isolates carried out using a one-compartment *in vitro* infection model.

The results of the multiple-isolate dose-ranging studies conducted in the one-compartment *in vitro* infection model are shown in Table 3. As evidenced by the high *r*^2^ of 0.897 for the relationship between change in log_10_ CFU/ml from baseline at 24 h and gepotidacin free-drug AUC/MIC ratio shown in Fig. 3, the data for the isolates pooled were well described by the Hill-type model. The Hill-type model parameter estimates (standard errors) were the following: *E*_0_, 2.33 (0.14); *E*_max_, 6.42 (0.27); Hill coefficient, 3.55 (0.50); and EC_50_, 40.4 (1.79). The median gepotidacin free-drug AUC/MIC ratios associated with net bacterial stasis and 1- and 2-log_10_ CFU reductions for the pooled data set were 33.9, 43.7, and 60.7, respectively.

**TABLE 3:** Summary of gepotidacin free-drug AUC/MIC ratios associated with bacterial reduction endpoints and Hill-type model parameter estimates^*a*^

Isolate	Magnitude of gepotidacin free-drug AUC/MIC ratio by bacterial reduction endpoint			Mean parameter estimates (SE) for Hill-type models				*r* ^2^
	Net bacterial stasis	1-log_10_ CFU/ml reduction	2-log_10_ CFU/ml reduction	*E*_0_	*E*_max_	EC_50_	*H*	
EC-13441	34.9	41.2	48.8	2.51 (0.21)	6.79 (0.47)	40.5 (2.15)	3.61 (0.66)	0.92
EC-ALL	34.9	44.4	55.0	2.40 (0.30)	8.58 (1.04)	53.7 (7.74)	2.19 (0.62)	0.95
EC-25922	32.8	53.4	83.0	2.62 (0.34)	9.22 (1.00)	82.6 (28.6)	1.00 (Fixed)	0.91
EC-IR5-3257	26.8	43.0	66.3	2.77 (0.46)	9.38 (1.78)	64.0 (35.4)	1.00 (Fixed)	0.84
Pooled value	34.5	41.3	49.7	2.33 (0.14)	6.42 (0.27)	40.5 (1.79)	3.55 (0.50)	0.90
Median value	33.9	43.7	60.7					

a The magnitude of gepotidacin free-drug AUC/MIC ratios associated with the bacterial reduction endpoints shown are based on Hill-type models describing the relationship between change in log_10_ CFU/ml and gepotidacin free-drug AUC/MIC ratio for individual and pooled E. coli isolates evaluated in the dose-ranging studies carried out using a one-compartment *in vitro* infection model. *E*_0_, change in log_10_ CFU/ml from baseline without drug; *E*_max_, maximal change in log_10_ CFU/ml from baseline; EC_50_, magnitude of free-drug AUC/MIC ratio associated with half-maximal effect; *H*, Hill coefficient; *r*^2^, coefficient of determination.

### Hollow-fiber *in vitro* infection model studies.

**FIG 4:**
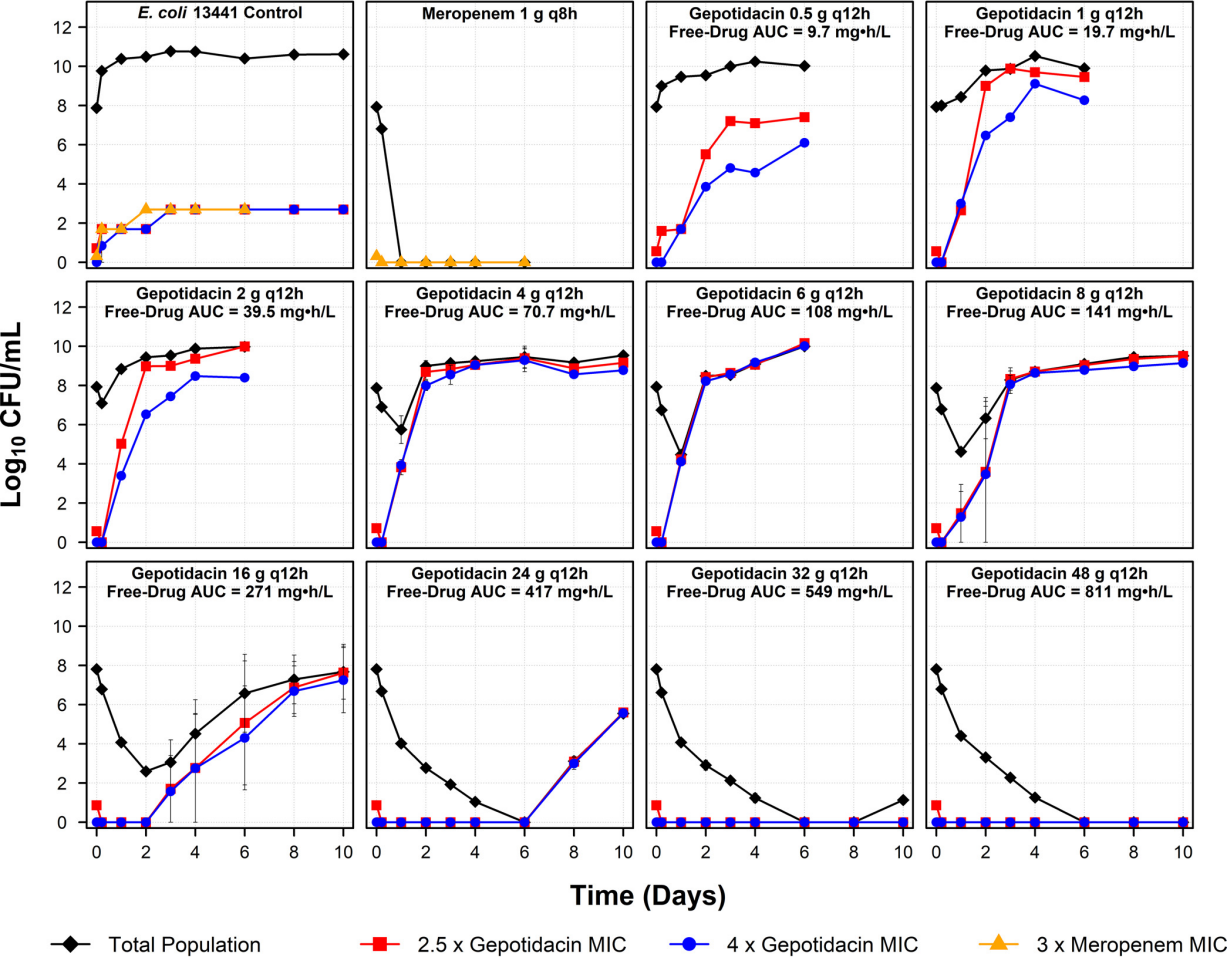
Total and antibiotic-resistant bacterial subpopulations over time for the no-treatment arm, meropenem at 1 g every 8 h (active, control arm), and gepotidacin at 0.5 to 48 g every 12 h based on data for E. coli NCTC 13441, evaluated using a hollow-fiber *in vitro* infection model.

The results of studies completed in the hollow-fiber *in vitro* infection model are presented in Fig. 4. The challenge isolate in the no-treatment control arm replicated well and reached a bacterial density of 2.5 × 10^10^ CFU/ml by study day 1. In the active control arm (meropenem), the total population (including the meropenem-resistant subpopulation) was driven to extinction by study day 1. Gepotidacin exposures, as represented by free-drug AUC/MIC ratios of 70.5 and less, amplified the drug-resistant subpopulation immediately, which often completely replaced the drug-susceptible subpopulation by study days 1 to 3. Gepotidacin free-drug AUC/MIC ratios of 136 to 209 markedly delayed the amplification of the drug-resistant subpopulation but ultimately failed to do so by study days 3 to 8.

**FIG 5:**
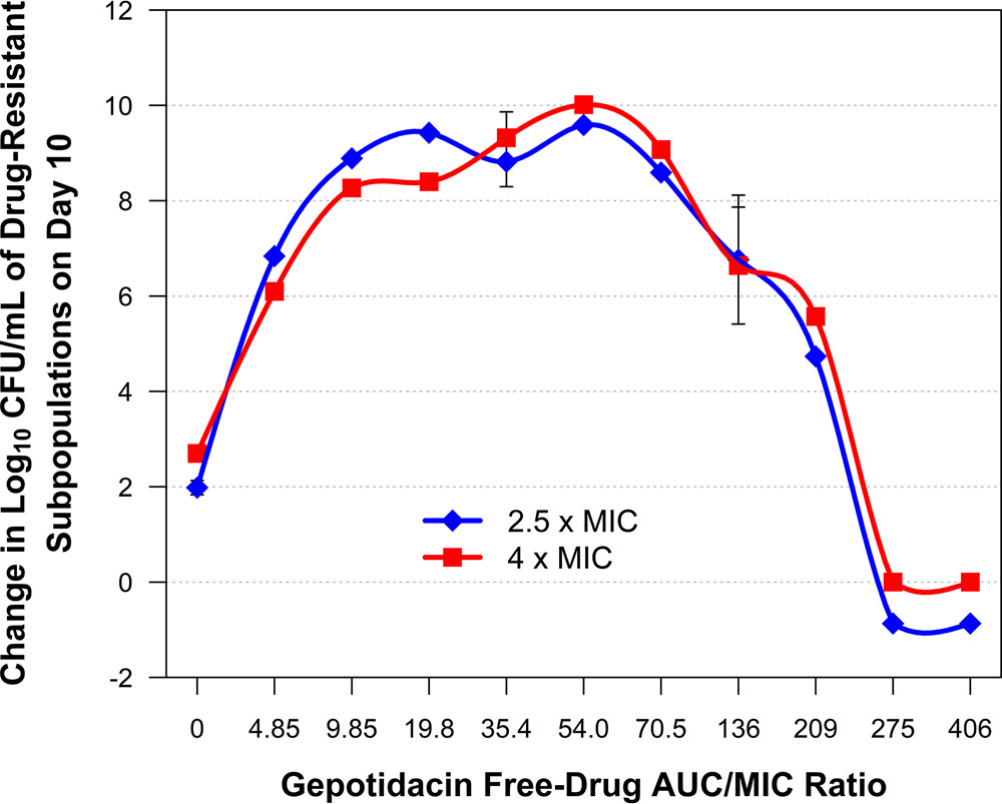
Relationships between change in log_10_ CFU/ml from baseline of the gepotidacin 2.5× and 4× MIC subpopulations on day 10 and gepotidacin free-drug AUC/MIC ratio based on data for E. coli NCTC 13441 evaluated using a hollow-fiber *in vitro* infection model.

**FIG 6:**
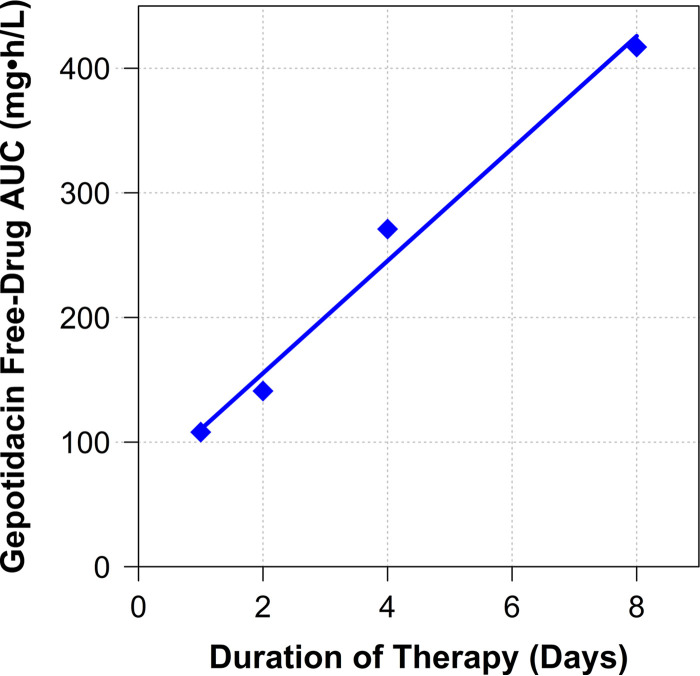
Relationship between gepotidacin free-drug AUC necessary to prevent amplification of the drug-resistant subpopulation to bacterial densities greater than that observed in the no-treatment control and therapy duration, based on data for E. coli NCTC 13441 evaluated using the hollow-fiber *in vitro* infection model.

The data presented in Fig. 4 were transformed to provide two additional figures, Fig. 5 and 6. Figure 5 shows the inverted-U relationships between change in log_10_ CFU/ml from baseline of the gepotidacin 2.5× and 4× MIC subpopulations on day 10 and gepotidacin free-drug AUC/MIC ratio. The gepotidacin free-drug AUC/MIC ratios of 275 and greater suppressed the amplification of the drug-resistant subpopulation for the entire study period.

Figure 6 shows the relationship between the gepotidacin free-drug AUC necessary to prevent amplification of the drug-resistant subpopulation to densities greater than observed in the no-treatment control and therapy duration. Note that as therapy duration increased, so too does the magnitude of the gepotidacin free-drug AUC necessary to prevent resistance amplification.

## DISCUSSION

The investigations carried out as described herein were designed to evaluate the conditions under which gepotidacin would likely be clinically effective for treating patients with E. coli infections. To this end, the studies undertaken had the following three objectives: (i) to identify the PK-PD index most closely associated with the efficacy of gepotidacin against E. coli; (ii) to determine the magnitude of the above-described PK-PD index associated with various bacterial reduction endpoints for E. coli; and (iii) to characterize the relationship between gepotidacin exposure and on-therapy resistance amplification. As described below, results of the studies conducted allowed for each of the above-described objectives to be achieved.

First, the dose-fractionation studies demonstrated that gepotidacin free-drug AUC/MIC ratio was the PK-PD index most closely associated with change in bacterial density of E. coli (Fig. 1). The PK-PD index associated with gepotidacin efficacy was the same as that demonstrated for fluoroquinolone agents (10). This observation was not unanticipated, as gepotidacin and fluoroquinolone agents bind to the same bacterial target enzymes (e.g., DNA gyrase and topoisomerase IV), although at different binding sites (5).

Second, dose-ranging studies conducted using multiple E. coli isolates allowed for determination of the magnitude of gepotidacin free-drug AUC/MIC ratio associated with various bacterial reduction endpoints for E. coli. As shown in Fig. 3, the relationship between the change in bacterial density and free-drug AUC/MIC ratio described the data for the four E. coli challenge isolates well. The median gepotidacin free-drug AUC/MIC ratios associated with net bacterial stasis and 1- and 2-log_10_ CFU reductions were 33.9, 43.7, and 60.7, respectively. While the magnitude of gepotidacin free-drug AUC/MIC ratio associated with net bacterial stasis was similar to that observed for fluoroquinolone agents, the gepotidacin free-drug AUC/MIC ratio associated with 1- and 2-log_10_ CFU/ml reductions from baseline were markedly lower. As shown by Andes and Craig, who studied gatifloxacin against *Enterobacteriaceae* in a neutropenic murine thigh infection model (11), the median gatifloxacin free-drug AUC/MIC ratios associated with net bacterial stasis and 1- and 2-log_10_ CFU/ml reductions were 40.7, 72.0, and 133, respectively.

Finally, the results of the studies conducted using a hollow-fiber *in vitro* infection model provided two important findings to consider when evaluating candidate gepotidacin dosing regimens for treatment studies. First, the relationship between gepotidacin exposure and resistance amplification assumed the form of an inverted U (Fig. 5), and second, as therapy duration increased, so too did the magnitude of gepotidacin exposure required to prevent resistance amplification (Fig. 6). For example, the gepotidacin free-drug AUC/MIC ratio needed to prevent resistance amplification for an 8-day treatment duration was nearly triple that for a 2-day regimen. The gepotidacin resistance mechanism identified herein was drug efflux, which resulted in a modest (2- to 4-fold) increase in MIC. This is important to note, as fluoroquinolone efflux has been shown to be a precursor to target site mutation(s) that confer high-level drug resistance (12). Essentially, gepotidacin drug efflux may allow for additional error-prone replication rounds and thereby increase the probability of a target site mutation(s) that would result in the loss of drug activity. Thus, to select a durable regimen, one must choose a gepotidacin dose (large enough) and therapy duration (short enough) that are associated with prevention of amplification of drug efflux mutants.

There were two limitations to the experiments and analysis described herein. The first limitation was that a single E. coli isolate was evaluated in the 10-day hollow-fiber *in vitro* infection model experiments. Thus, it was not possible to evaluate the interisolate variability associated with the gepotidacin exposure required to prevent the amplification of resistant subpopulations. The second limitation was the hollow-fiber infection model is an *in vitro* system and, as such, the effect of the immune system on the activity of gepotidacin could not be evaluated.

In conclusion, we successfully characterized the conditions under which gepotidacin is expected to be effective against E. coli. Specifically, we identified the free-drug gepotidacin AUC/MIC ratio as the PK-PD index associated with efficacy, determined the magnitude of free-drug AUC/MIC ratio associated with various bacterial reduction endpoints for E. coli based on a challenge panel of E. coli isolates, and characterized the relationship between gepotidacin exposure and on-therapy resistance amplification. Together, the data generated from these studies will be useful to support discrimination among candidate gepotidacin dosing regimens for future clinical study.

## MATERIALS AND METHODS

### Bacteria and antimicrobial.

Four E. coli isolates were utilized throughout all *in vitro* model and susceptibility testing studies. Two isolates were provided by GlaxoSmithKline (GSK; Collegeville, PA), and two were purchased as reference strains from the American Type Culture Collection (ATCC; Manassas, VA) and National Collection of Type Cultures (NCTC; Public Health England). Gepotidacin powder was provided by GSK, while levofloxacin and meropenem were purchased from Henry Schein Medical (Melville, NY).

### (i) Media and *in vitro* susceptibility studies.

Susceptibility studies, using cation-adjusted Mueller-Hinton II (MHII) broth and Mueller-Hinton (MH) agar (Becton, Dickinson and Company, Franklin Lakes, New Jersey), were completed in triplicate per the Clinical and Laboratory Standards Institute (CLSI) guidelines (13). Gepotidacin, meropenem, and levofloxacin susceptibility studies were conducted using broth microdilution and agar dilution methodologies. E. coli ATCC 25922 was utilized as an internal standard for MICs based on CLSI guidelines (14).

### (ii) Frequency of resistance studies.

The frequency of resistance was estimated by plating 2 ml of log-phase growth suspension onto MH agar medium supplemented with 2.5 and 4 times the baseline gepotidacin agar MIC and was performed over two separate trials. The bacterial concentration within the suspension was determined by quantitative culture. The ratio of growth found on the drug-containing plates to that of the starting inoculum provided an estimate of the drug resistance frequency within a total population. A subset of isolates was collected from the drug-containing plates for evaluation of susceptibility to gepotidacin and tested for a change in the MIC from the baseline to confirm decreased susceptibility using the agar dilution method described above. To evaluate the role of efflux pumps on the susceptibility of isolates collected from the drug-supplemented agar plates, gepotidacin MIC values were also determined using agar supplemented with 40 mg/liter broad-spectrum efflux pump inhibitor Phe-Arg beta-naphthylamide dihydrochloride (Sigma, Aldrich, St. Louis, MO).

### (iii) One-compartment *in vitro* infection model.

The one-compartment *in vitro* infection model utilized in these studies has been extensively utilized for the evaluation of the PK-PD of antimicrobials and has been previously described (15–18). Briefly, the *in vitro* model is composed of a central infection compartment containing bacterial growth medium, the isolate of choice, and a magnetic stir bar to ensure homogeneity. The central infection compartment was attached to a stir plate, set to a speed of 125 rpm, and housed within a temperature- and humidity-controlled incubator set at 35°C. Using peristaltic pumps, drug-free MHII broth medium is infused into the central infection compartment while simultaneously being removed through an exit port and captured in a waste container to simulate a desired pharmacokinetic (PK) profile. The central compartment is inoculated with the organism of choice and then exposed to concentration-time profiles representing human free-drug exposures of gepotidacin (19), administered orally every 12 h (q12h), assuming 33% protein binding. The test compound was infused into the central compartment using computer-controlled syringe pumps to simulate the desired dosing frequency, duration, and concentrations. Specimens for CFU enumeration and drug concentration assay were collected from the central infection compartment at predetermined time points.

E. coli bacterial suspensions, at a burden of 1.0 × 10^6^ CFU/ml, were prepared from overnight cultures grown from frozen stock vials on Trypticase soy agar (TSA) supplemented with 5% sheep blood (BD Laboratories). Isolates were taken from the overnight cultures, placed within a 125-ml Erlenmeyer flask containing 30 ml of MHII broth, incubated at 35°C, and mixed at 125 rpm. The bacterial concentration within the flask was determined by optical density utilizing a previously confirmed growth curve specific to each E. coli suspension. The bacterial suspensions within the central compartment were then exposed to various gepotidacin concentrations, which simulated human free-drug concentration-time profiles for a range of exposures described below.

Samples (1 ml) were collected from the central compartment for the determination of bacterial burden at 0, 2, 4, 8, 12, and 24 h. Each bacterial sample was centrifuged, the supernatant discarded, and the bacterial pellet washed and resuspended with sterile normal saline twice to prevent drug carryover. The bacterial suspensions were then cultured onto TSA plus 5% sheep blood agar plates as well as MH agar supplemented with 2.5 and 4 times the gepotidacin agar MIC. All plated bacterial samples were incubated at 35°C for 24 h within a humidified incubator. One-milliliter specimens for drug assay were collected over various time points throughout the 24-h experiment and then immediately frozen at –80°C until being assayed for drug concentration.

### (iv) Dose-fractionation studies.

The isolate chosen for dose-fractionation studies, E. coli NCTC 13441, was chosen based on the reproducibility of its results as well as its representation of the ST-131 clonal group. Seven total daily gepotidacin exposures, as measured by free-drug AUC over 24 h, were held constant but fractionated into doses administered every 6, 12, or 24 h (q6h, q12h, and q24h, respectively). One-milliliter samples were removed from the central compartment for CFU determination at 0, 2, 4, 8, 12, and 24 h. Every sample collected for the enumeration of bacterial burden was centrifuged, washed, and resuspended with sterile normal saline twice to prevent drug carryover and then cultured onto TSA plus 5% sheep blood agar plates. All plated cultures were then incubated at 35°C for 24 h. Over the 24-h experiment, samples for the evaluation of gepotidacin concentration-time profiles were collected at various time points. All 1-ml samples were immediately frozen at –80°C until being assayed via liquid chromatography-tandem mass spectrometry (LC-MS/MS) for determination of unknown concentrations.

### (v) Gepotidacin dose-ranging studies.

Using the one-compartment *in vitro* infection model, a series of 24-h dose-ranging studies were conducted to evaluate the relationship between gepotidacin exposure and bactericidal activity. In these studies, four E. coli isolates were subjected to a range of gepotidacin exposures, administered q12h. All studies were completed in duplicate and compared to a no-treatment control. Samples were collected for pharmacokinetics and CFU determination using the methods described above.

### Hollow-fiber *in vitro* infection model.

The utilization of the hollow-fiber *in vitro* infection model to identify exposures required to prevent amplification of resistant subpopulations has been utilized for many years and is supported by the EMA (20) and FDA (21) guidelines for evaluation of any new antibiotic. The hollow-fiber *in vitro* infection model utilized in the studies described herein has been previously described (22, 23). In brief, this pharmacodynamic *in vitro* system supports the growth of pathogens within the peripheral compartment of the hollow-fiber cartridge made up of semipermeable membranes. These membranes enclose the bacterial suspension while simultaneously allowing nutrients, drugs, and bacterial metabolites to transverse freely into and out of the peripheral compartment. To propagate the bacterial cultures over an extended period of time, fresh medium is circulated from the central compartment through the hollow-fiber cartridge using peristaltic pumps. The challenge compound of choice is pumped into the central compartment, under computer control, and is continually diluted in the central compartment to simulate any given half-life. Due to the high surface area-to-volume ratio, of the thousands of membranes within the hollow-fiber cartridge, drug concentrations rapidly equilibrate within the enclosed system. Sample ports located on the hollow-fiber cartridge allow for sampling of quantitative cultures and drug concentration-time profiles throughout the study duration.

Using the hollow-fiber *in vitro* infection model studies described herein, a single E. coli isolate (NCTC 13441) at an initial bacterial burden (10 ml of 1.0 × 10^8^ CFU/ml) was challenged by gepotidacin free-drug AUC/MIC ratios ranging from 4.85 to 406, administered as q12h doses. Gepotidacin free-drug AUC values over 24 h were determined using the same PK parameters utilized in the one-compartment *in vitro* infection model studies. These regimens were chosen to achieve a wide range of effect from treatment failure (growth matching that of the no-treatment control at 24 h) to that associated with near maximal effect (at or near sterilization). To compare the activity of gepotidacin to that of an active clinical dosing regimen, free-drug concentration-time profiles of meropenem representing a 1-g q8h regimen administered over a 0.5-h infusion (24) were simulated in the system. Each active and inactive treatment regimen was evaluated in duplicate over a 10-day period, requiring 24 hollow-fiber bioreactors in total.

Samples for the determination of bacterial burden were collected for each regimen at initiation of treatment and 5 h postinitiation as well as on days 1, 2, 3, 4, 6, 8, and 10. All bacterial samples were centrifuged and washed to eliminate drug carryover as described previously. The total bacterial burden was determined by plating a portion of the washed culture onto TSA plus 5% sheep blood agar plates, and the presence of a drug-resistant subpopulation was evaluated through the use of MH agar plates supplemented with gepotidacin concentrations representing 2.5 or 4 times the agar MIC value. All plated samples were placed in a humidified incubator for a 24-h period at 35°C, after which the bacterial burden was determined. Samples for the determination of simulated concentration-time profiles for each active treatment regimen were collected over the first 48 h.

### (i) Analytical method.

All samples were assayed by LC-MS/MS with an AB Sciex API5000 mass spectrometer (Framingham, MA). The standard curves for gepotidacin and meropenem were linear for both compounds, with values ranging from 0.05 to 80 mg/liter and 0.1 to 100 mg/liter, respectively. The lower limit of quantitation was 0.05 and 0.1 mg/liter for gepotidacin and meropenem, respectively.

### (ii) Pharmacokinetic-pharmacodynamic analysis.

PK models were fit to the PK samples collected for the evaluation of the drug concentration profile. The gepotidacin free-drug AUC and maximum gepotidacin concentration (*C*_max_) over 24 h, and the proportion of time over 24 h that free-drug concentrations were above gepotidacin MIC values (%*T*>MIC) was calculated. These PK-PD indices were evaluated using data from the dose-fractionation and dose-ranging studies conducted using the one-compartment *in vitro* infection models as described below.

To identify the PK-PD index associated with the efficacy of gepotidacin, data from the 24-h one-compartment *in vitro* infection model dose-fractionation studies were evaluated using Hill-type models and nonlinear least-squares regression. All data were weighted using the inverse of the estimated measurement variance. The relationships between change in log_10_ CFU/ml from baseline at 24 h and free-drug AUC/MIC ratio, *C*_max_/MIC ratio, and %*T*>MIC were evaluated.

To evaluate the interisolate variability surrounding the PK-PD index associated with gepotidacin activity, the data from the multiple-isolate dose-ranging studies were evaluated using the same Hill-type models described above. Using the PK-PD index that was most closely associated with efficacy, as identified based on the results of the above-described dose-fractionation studies, the magnitude of this PK-PD index associated with net bacterial stasis and 1- and 2-log_10_ CFU/ml reductions from baseline at 24 h were calculated for each individual isolate as well as for the pooled data set.
